# Influence of nitrogen fertilization, seed inoculation and the synergistic effect of these treatments on soybean yields under conditions in south-western Poland

**DOI:** 10.1038/s41598-024-57008-y

**Published:** 2024-03-20

**Authors:** Magdalena Serafin-Andrzejewska, Anna Jama-Rodzeńska, Waldemar Helios, Marcin Kozak, Sylwia Lewandowska, Dariusz Zalewski, Andrzej Kotecki

**Affiliations:** 1 Institute of Agroecology and Plant Production, Wrocław University of Environmental and Life Sciences, Grunwaldzki Sq. 24 A, 50-363 Wrocław, Poland; 2 Department of Genetics, Plant Breeding and Seed Production, Wrocław University of Environmental and Life Sciences, Grunwaldzki Sq. 24 A, 50-363 Wrocław, Poland

**Keywords:** Inocultation, Nitrogen fertilization, Seed yield, Straw yield, Yield components, Yield elements, Genetics, Plant sciences, Ecology

## Abstract

Soybean, belonging to legumes, has a specific ability to biological nitrogen fixation, which can be reinforced by seeds inoculation. However, support with a starter dose of mineral nitrogen fertilizer may be necessary to achieve high seed yields. A four-year field experiment was conducted to determine the effect of mineral N fertilization (0, 30, 60 kg ha^−1^), seed inoculation with two commercial inoculants and combinations of these treatments on yield components and yielding of soybean in conditions of south-western part of Poland. The synergistic effect of mineral fertilization at dose 30 kg ha^−1^ and inoculation on soybean productivity was the most beneficial. Similar effects were observed when 60 kg N ha^−1^ was applied both separately and with inoculation. However, due to the environmental impact of mineral fertilizers and to promote plants to biological nitrogen fixation (BNF), it is advisable to use lower doses of N fertilizer (at 30 kg ha^−1^) and inoculate soybean seeds in agro- climatic conditions of south-western Poland. Therefore, based on this study we recommend to apply starter dose of N and inoculation.

## Introduction

Soybean (*Glycine max* (L.) Merr.) is one of the most important legumes crops cultivated in the world. Since 1990, world production of soybean has increased mainly due to increased production of this species in North and South American countries. Such countries as USA, Brazil, Argentina are the greatest producers of soybean in the world^1^. In Poland, soybean has been grown for about 140 years, however, significant progress in its cultivation appeared in the second decade of the twenty-first century. In Poland a limiting factor for soybean is cultivar progress related to the adaptation of cultivars to the country’s climate and soil conditions, taking into account the length of vegetation season and available sunlight and temperature^2^.

In Poland, as well in EU, there is a need to provide non-GMO feed protein source. In EU soybean meal is used as the primary source of plant protein in the feeding for livestock, i.e., poultry and cattle^3^. The European Union (EU) is the largest importer of soybean meal that accounts for 70% of the high-protein components used in the production of compound feeds. Therefore, Europe's large imports of soybean meal have resulted in a dependence on protein imports. Therefore, attempts to soybean cultivation in Polish conditions seems to be promising. One way to achieve protein balance stability is to improve legume yields and, in particular, to improve soybean agronomics in European countries^2,4–9^.

Average yields in Poland depend on the cultivation region and cultivar range from 2.5 to 3 t per ha^10^. The area under soybean cultivation in 2023 amounted 44 621 ha^11^. Research to date has shown that very early, early and mid early cultivars available on the domestic seed market can be grown without any major risk in all regions of Poland, while from the late and very late group are suitable for cultivation mainly in the southern and partly in the central regions of the country^12^.The application of new technologies with low carbon emissions is necessary for sustainable agriculture^13,14^. Therefore, practices that minimize and/or optimize input use should be applied in agricultural systems^15^.

The yield and quality of soybean depends on soil, weather and agrotechnical conditions, including mineral fertilization and inoculation of seeds. Nitrogen fertilization and seed inoculation are the main factors affecting the yield and chemical composition of soybean seeds. As a legume, soybean can fix free atmospheric nitrogen due to symbiosis with bacteria *Bradyrhizobium japonicum*. Becuase of this phenomenon, soybean is able to supply the soil up to 100 kg ha^−1^ of N contributing to the lowering nitrogen fertilization^16^. Soybean is characterized by high nitrogen (N) requirements and therefore needs to take up 80 kg of N per Mg of seed production^17^. On average, 50–60% and up to 90% of nitrogen is supplied through biological nitrogen fixation (BNF) by symbiotic soil bacteria^18^. Atmospheric nitrogen is converted to the plant-available ammonium form affecting the growth, development and yield of soybean^19^. To obtain high seed yield of soybean and provide the nitrogen (N) requirements of this crop, biological N fixation must be performed at its maximum efficiency^15,20^. However, *B. japonicum* is not found in Central European soils, therefore soybean seeds are inoculated with these bacteria prior to sowing^21^. Inoculation of soybean is a common and widespread practice in Europe, Australia and America, where many successes have been reported and allows to improve productivity of this species^22–26^. This is a much more common management practice in South America than in the United States (USA). In Brazil and Argentina, about 80% of soybean fields are inoculated annually, while in the U.S. only 15%^27^. Seed inoculation is a cost-effective practice with economic benefits. Various inoculation methods can be used. Inoculation of soybeans is usually done by coating the seeds with symbiotic bacteria before sowing. The success of *Rhizobium* inoculation depends primarily on the type of rhizobia, environmental conditions, and crop cultivation^5^. Therefore, it is common to treat seeds with appropriate bacteria, thereby reducing the use of mineral nitrogen fertilization^28^. Symbiosis is able to cover soybean's nitrogen requirements in the range of 30% to 60%^29^. The relationships between seed inoculation, nitrogen metabolism, yield and seed composition are a complex problem in soybean cultivation.

The aim of the study was to evaluate the effect of various doses of nitrogen, inoculation and synergistic effect of these treatments on the yield and yield components of soybean. In the research hypothesis we assume that seed inoculation or synergistic effect of inoculation and nitrogen fertilization will be more beneficial for soybean productivity than sole fertilization. Such result would be consistent with sustainable agriculture management.

## Materials and methods

### Experimental setup and design

The study was conducted in 2016–2019 at the experimental fields of Wroclaw University of Evironmental and Life Sciences (51° 111′ N, 17° 85′ E) in Poland. A four-year two-factor field experiment was established in four replications using randomized complete block design (RCBD). Studied factors:

- Bacterial inoculant: control, Inoculant 1, Inoculant 2
- N fertilization: 0, 30, 60 kg ha^−1^

### Agrotechnical management

Winter wheat served as the previous crop. After harvest, stubble ploughing was applied at the depth of 8 cm. Next pre-winter ploughing in the depth 25 cm was carried out. Harrowing was done in spring and pre-sowing P and K fertilization was performed. Phosphorus was used as triple superphosphate (40%) at the dose 60 kg ha^−1^ P_2_O_5_ and potassium was used as 60% sylvinite in the amount 120 kg ha^−1^ K_2_O. According to experiment design nitrogen fertilization was applied in doses: 30 and 60 kg ha ^−1^ (ammonium nitrate 34%). All fertilization was done before sowing.

Annushka (non-GMO, Ukrainian breeding) was used as tested cultivar in field experiment. It is a very early cultivar with the length of the growing season about 100–130 days. Potential seed yield is up to 4 t ha^−1^, with high protein and oil contents. 1000-seed weight ranged from 110 to 155 g. Protein amount in seeds is 36–40% and oil content 17–21%. The detailed information about seed germination and sowing rate are given in the Table 1.

**Table 1:** Sowing rate and seed germination in years of research (2016–2019).

Parameter	2016	2017	2018	2019
Sowing rate (kg ha^−1^)	152	133	140	162
Seed germination (%)	92	95	93	89

Each inoculant was applied on seeds directly before sowing, according to the manufacturer's recommendations. **Inoculant 1** is used in soybean cultivation. It contains live bacteria cultures of the *Rhizobium* group (*Bradyrhizobium japonicum*). This product contains at least 2 × 10^9^ (at least 2 billion) live bacteria (*B. japonicum*) for use in soybean cultivation per gram of peat substrate. To the peat substrate, original polymer was added at a low concentration to ensure adhesion and safety. **Inoculant 2** contains live papillary bacteria of the genus *Bradyrhizobium* capable of fixing free atmospheric nitrogen in symbiosis with legumes. As a carrier for the nodule bacteria perlite was used. Standard content of live bacteria is 10^8^ (one houndred milion). The inoculants certainly differed in their composition, i.e. the strains of bacteria within *Bradyrhizobium japonicum.*

Soybeans were sown in the third week of April each year at a density of 90 plants per m^2^ at the depth 3–4 cm and the row spacing was 15 cm. Plant protection was carried out according to integrated pest management. Weed control was performed as given in Table 2. Pest and fungal disease were not observed, so insecticides and fungicides were not used.

**Table 2:** Herbicides used in soybean cultivation in the years of research.

2016	2017	2018	2019
Linurex 500SC − 1 dm^3^ ha^−1^ (linuron)	Boxer 800 EC − 4,0 dm^3^ ha^−1^ (prosulfocarb)	Boxer 800 EC − 4,0 dm^3^ ha^−1^ (prosulfocarb)	Boxer 800 EC − 4,0 dm^3^ ha^−1^ (prosulfocarb)
Corum 502,4 SL − 1,25 dm^3^ ha^−1^ + Dash HC 0,6 dm^3^ ha^−1^ (bentzone + imazamox and adiuvant)	Select Super 125 EC − 2,0 dm^3^ ha^−1^ (clethodym)	Corum 502,4 SL − 1,25 dm_3_ ha^−1^ + Dash HC 0,6 dm^3^ ha^−1^ (bentzone + imazamox and adiuvant)	Corum 502,4 SL − 1,25 dm^3^ ha^−1^ + Dash HC 0,6 dm^3^ ha^−1^ (bentzone + imazamox and adiuvant)
	Corum 502,4 SL − 1,25 dm^3^ ha^−1^ + Dash HC 0,6 dm^3^ ha^−1^ (bentzone + imazamox and adiuvant)	Fusilade Forte 150 EC − 1,5 dm^3^ ha^−1^ (fluazifop-P-butyl)	Targa Super 05 EC − 2,5 dm^3^ ha^−1^ (chisalofop-P-ethyl)

The seeds were harvested in the second/third week of September at full maturity.

### Soil conditions

The experiment was set up on typical brown luvisol developed from light loam underlain by medium loam^30^, rated as class IIIb (3rd class of 9) in Poland, suitable for wheat cultivation^10^. Contents of macronutrients and pH in the soil are shown in Table 3 and are as follows: medium to very high P content, medium K content, medium to high Mg content and slightly acidic pH of soil^31^.

**Table 3:** Macronutrients in soil (mg kg^−1^) and pH of soil in 2016–2019.

Years	pH in 1 M KCl	P	K	Mg
2016	5.7	93	157	64
2017	5.9	111	154	101
2018	6.5	62	145	82
2019	5.9	63	161	90

### Weather conditions

**Figure 1:**
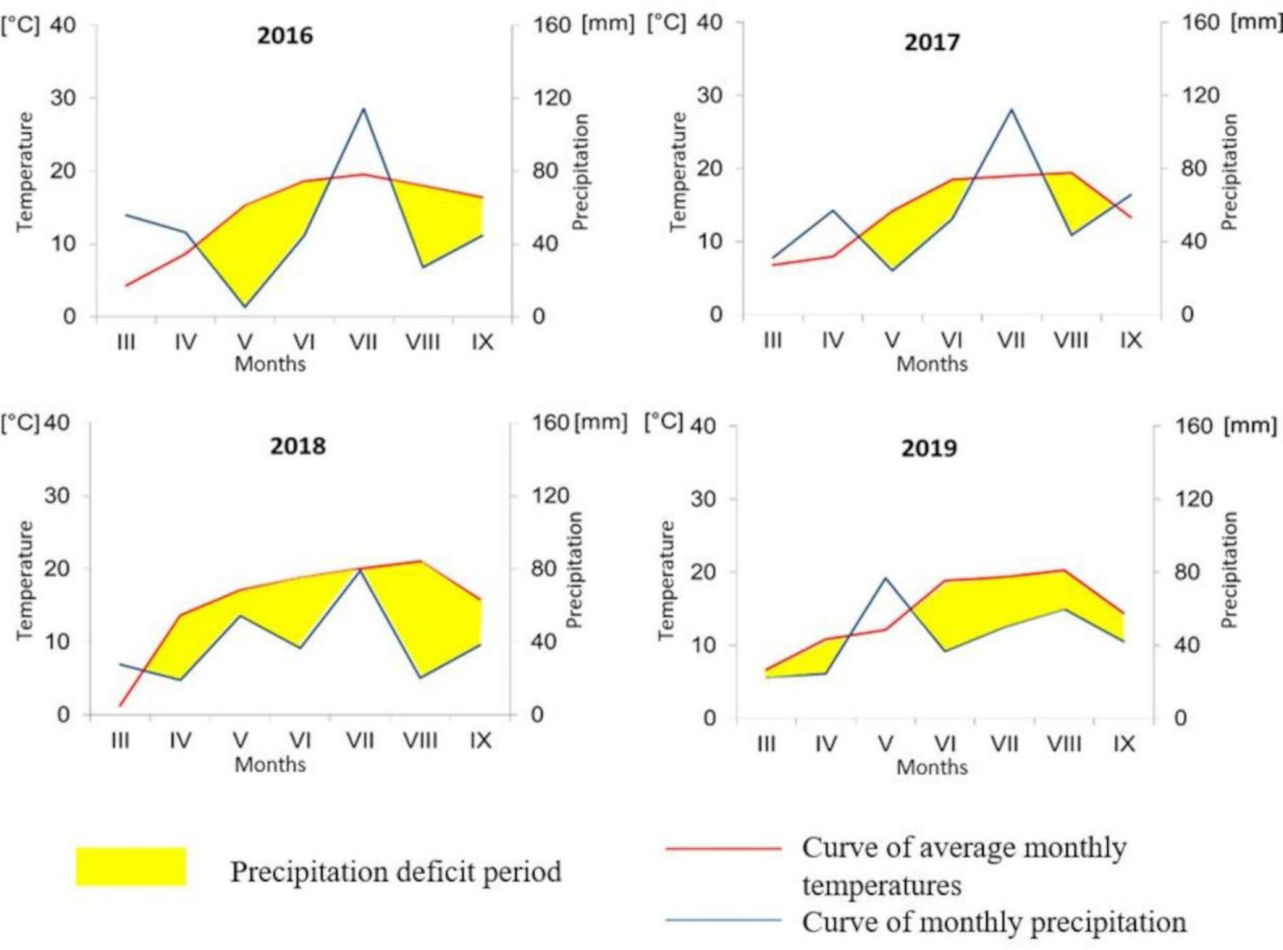
Weather conditions (water deficiency) in the years of experiment according to Gaussen-Walter diagrams modified by Łukasiewicz (2006)^32^ . Roman numerals on the x-axis are the months.

Weather conditions are presented at the Gaussen-Walter diagrams, modified by Łukasiewicz (2006)^32^. They were variable during the study years and had a significant impact on the course of vegetation and soybean yield. On these diagrams 1 °C corresponds to 4 mm of precipitation (Fig. 1). Roman numerals on the x-axis are the months. The Gaussen-Walter diagram directly represents data concerning only temperature and precipitation levels. It is also possible to get the information on the amount of evapotranspiration, and thus estimate the excess or deficiency of precipitation.

### Crop yield and agronomic data collection

Ten plants were taken randomly from each plot to determine the components of the yield. The components of yield included: number of pods per plant, number of seeds per plant, weight of 1000 seeds (g). Plant density from 1m^2^ was done before harvesting. The seed and straw yield from plots was converted to yield per hectare at 15% moisture content. The area of a single plot was 15 m^2^. The results were presented as an average value.

### Statistical analysis

According to the central limit theorem, the obtained results were assumed to have a normal distribution. The Levene’s test was used to estimate the homogeneity of variances. The analysis of variance (ANOVA) was performed at a significance level of *p* < 0.05 using Statistica program 13.1 (StatSoft, Kraków, Poland)^33^. Due to significant effect of the weather conditions during this study on the tested traits, *p-values* for factors and their interactions are presented for each year separately. But *p-value* is also presented for measured traits as an average for four years of study, where years of research are statistically factor. Homogeneous groups were determined by Tukey’s multiple range test using consecutive letters starting from “a”—the highest to “g”—the lowest value in terms of analyzed traits. Data Mining (variable selection and elimination) was used to plot the importance of the dependent variables. The GLM (general linear model) was used to prepare the data for statistical analysis.

Continuous effects were marked as follows:

- Plant density, number of pods per plant, number of seeds per plant, weight of 1000 seeds, straw yield.
- Quality effects: years, inoculation, fertilization.
- Covariate: plant density, number of pods per plant, number of seeds per plant, weight of 1000 seeds, straw yield.

## Results

### Statistical analysis

**Figure 2:**
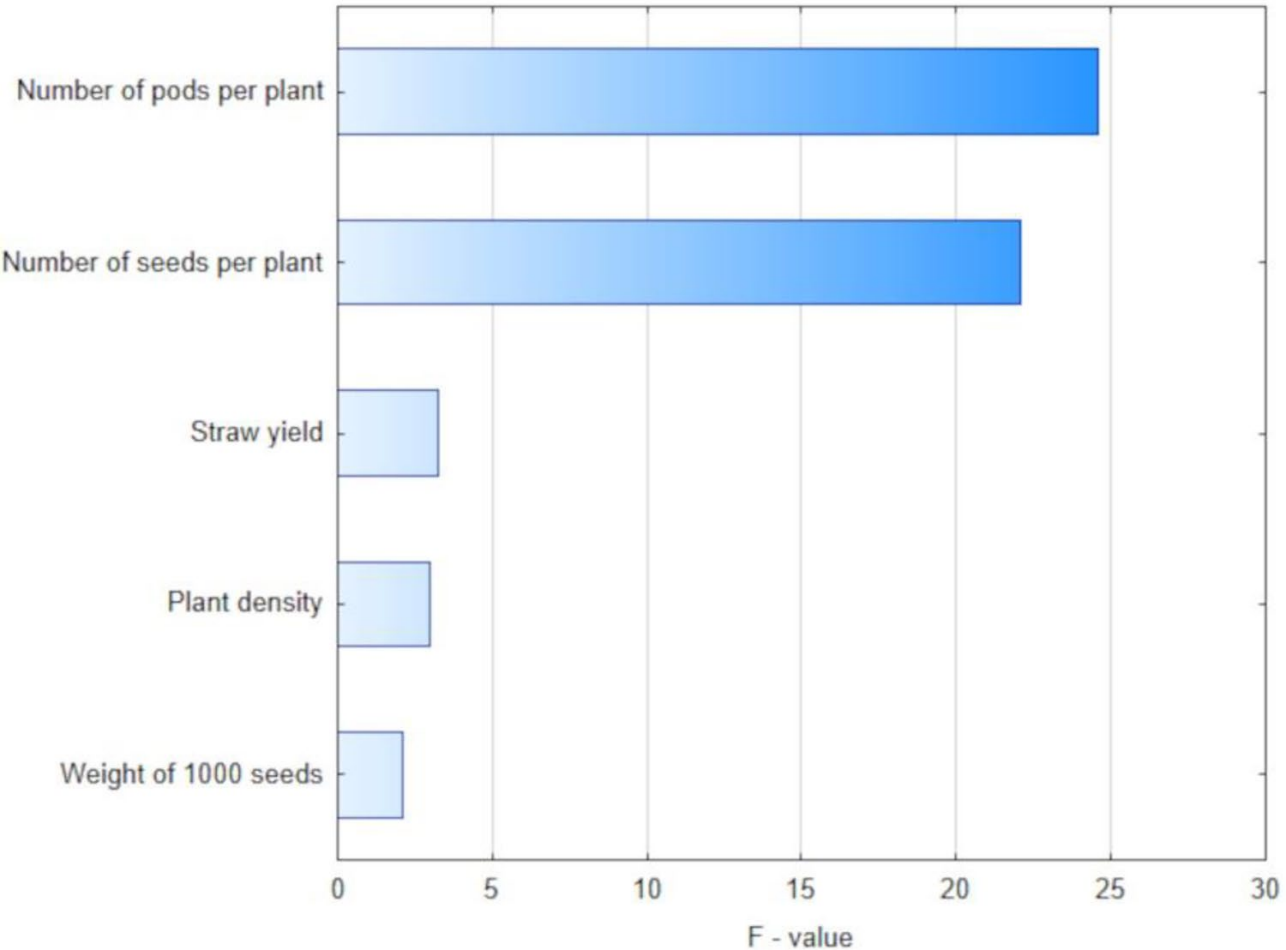
Importance of quantitative predictors of seed yield (2016–2019).

The most important yield components, on which soybean seed yield depends are plant density before harvest, number of pods per plant, number of seeds per pod and 1000-seed weight. The product of the values of these traits gives potential yield of soybean seeds. Figure 2 presents the effect of examined traits on the seed yield of soybean. According to the F-value, the number of pods per plant had the greatest impact on seed yield while plant density and weight of 1000 seeds – the lowest.

All examined traits were stastistically dependent on the weather conditions in the years of research (Table 4). Number of pods per plant as well as number of seeds per plant were statistically related to all tested factors and their interaction. Weight of 1000 seeds was stastistically connected with the weather conditions in the years of research as well as inoculation (Table 4).

**Table 4:** The effect of fertilization and inoculation on the yield components (for four years of research 2016–2019) – Three-way ANOVA.

Source	Plant density (pcs m^−2^)	Number of pods per plant (pcs)	Number of seeds per plant (pcs)	Weight of 1000 seeds (g)
*P*- value				
Years	0.0000	0.0000	0.0000	0.0000
Inoculation	0.0733	0.0000	0.0000	0.0011
Fertilization	0.9049	0.0000	0.0000	0.7332
Years × Inoculation	0.1754	0.0000	0.0000	0.4975
Years × Fertilization	0.9399	0.0000	0.0000	0.4336
Fertilization × Inoculation	0.1995	0.0000	0.0000	0.9299
Years × Fertilization × Inoculation	0.4721	0.0000	0.0000	0.9063

Seeds and straw yield were stastistically dependent on all examined factors and their inteactions, except one observation. Interaction of fertilization and inoculation did not affect seed yield (Table 5).

**Table 5:** The effect of fertilization and inoculation on seeds and straw yield (for four years of research 2016–2019) – Three-way ANOVA.

Source	Yield (t ha^−1^)	
	Seeds	Straw
*P*- value		
Years	0.0000	0.0000
Inoculation	0.0221	0.0000
Fertilization	0.0008	0.0000
Years × Inoculation	0.0003	0.0000
Years × Fertilization	0.0184	0.0235
Fertilization × Inoculation	0.1026	0.0012
Years × Fertilization × Inoculation	0.0176	0.0045

Due to the strong dependence of soybean plant development and yield on variable weather conditions during the study years, the results are also presented as a statistical analysis for each year separately.

While thermal conditions were favourable for soybean growth in 2016, 2017, 2018 and 2019, moisture conditions were unfavorable in 2016 (defict water in May shortened the length of the vegetative period in favor of the generative one), 2017 and 2018 (combinations of water defict with high temperature led to shortening the phase of pod development and faster drying up the plants before harvest). In 2019 lower precipitation and high temperature during pods ripening caused higher values of yield components (and there was no spontaneous shattering of pods) what suggests that soybean is drough resistant due to its morphological structure and heliotropic adjustments of leaflet angles (Fig. 1). In 2016 all examined factors and their interaction had significant impact on number of pods per plant and number of seeds per plant. Inoculation and synergistic effect of tested factors affected plant density in 2017. Nitrogen fertilization, inoculation and interaction affected the number of seeds per plant, while fertilization and inoculation impacted the number of pods per plant. Inoculation had significant impact on weight of 1000 seeds in 2017. In 2019 only fertilization had impact on number of pods per plant and number of seeds per plant (Table 6).

**Table 6:** The effect of fertilization and inoculation on the yield components for each year of experiment – Two-way ANOVA.

Source	*P*- value			
	2016	2017	2018	2019
Plant density (pcs m^−2^)				
Fertilization	0.9292	0.8400	0.7792	0.7017
Inoculation	0.1679	0.0013	0.7389	0.5820
Fertilization × inoculation	0.4491	0.0241	0.8284	0.2917
Number of pods per plant (pcs)				
Fertilization	0.0000	0.0000	0.0797	0.0003
Inoculation	0.0000	0.0000	0.3895	0.9144
Fertilization × inoculation	0.0000	0.0629	0.7809	0.7766
Number of seeds per plant				
Fertilization	0.0000	0.0001	0.4348	0.0013
Inoculation	0.0000	0.0000	0.7398	0.2888
Fertilization × inoculation	0.0000	0.0299	0.6511	0.1116
Weight of 1000 seeds (g)				
Fertilization	0.0692	0.9425	0.7921	0.5976
Inoculation	0.1057	0.0258	0.1768	0.8334
Fertilization × inoculation	0.6250	0.9719	0.9792	0.4370

The effect of investigated factors and their interaction on seeds and straw yields varied in the years of research. In 2016 all examined factors and their interaction had significant impact onseed and straw yield. In 2017 inoculation influenced on seeds yield. In 2019 inoculation and interaction of studied factors had impact only on straw yield. (Table 7).

**Table 7:** The effect of fertilization and inoculation on seeds and straw yield for each year of experiment – Two-way ANOVA.

Source	*P*- value			
	2016	2017	2018	2019
Seeds yield (t ha^−1^)				
Fertilization	0.0000	0.0715	0.2028	0.3770
Inoculation	0.0000	0.0043	0.2823	0.2102
Fertilization × inoculation	0.0000	0.9181	0.2584	0.8043
Straw yield (t ha^−1^)				
Fertilization	0.0001	0.4382	0.8640	0.0816
Inoculation	0.0001	0.6422	0.9198	0.0016
Fertilization × inoculation	0.0036	0.8920	0.4846	0.0011

### Mean values of examined features based on two-way ANOVA

The effects of fertilization and inoculation on the most important yield components for soybeans, as plant density before harvest, number of pods per plant, number of seeds per plant, as well as 1000 seed weight, are described below. For more detailed information, please see Supplementary Tables S1-S4.

Only in 2017 the effect of tested factors and their interaction on plant density was observed. The highest plant density was found while using Inoculant 2. Synergy of Inoculant 2 and N fertilization with 30 kg N ha^−1^ was the most beneficial (Supplementary Tab. S1). Despite significant differences in plant density in 2017, the value of this trait was the lowest compared to other years of research.

The number of pods per plant in 2016 and 2017 was the highest when Inoculant 1 was applied (Supplementary Tab. S2). In 2016 and 2019 the highest value of number of pods per plant was observed under N fertilization with 60 kg ha^−1^ whereas in 2017 with doses 30 and 60 kg ha^−1^. In 2016 this component of the yield was the most beneficial under effect of Inoculant 1 combined with 30 kg ha^−1^, and when only N fertilization with 60 kg ha^−1^ was used, without inoculation (Supplementary Tab. S2).

Inoculant 1 had significant impact on the number of seeds per plant in 2016 and 2017 (Supplementary Tab. S3). Nitrogen fertilization affected differently this trait in the years of research. In 2016 and 2019 dose of 60 kg ha^−1^ turned out to be the most effective, while in 2017 both tested doses: 30 and 60 kg ha^−1^. Taking into account the interaction of factors in two first years of research Inoculant 1 and N fertilization at 30 kg ha^−1^ was the most beneficial, but in 2017 Inoculant 1 and 60 kg ha^−1^ gave statistically comparable effect.

Weight of 1000 seeds did not depend on tested factors in the years of research except for 2017, when Inoculant 1 caused increase of this trait (Supplementary Tab. S4).

Seed yield is the most important quantitative trait related to the economic efficiency of soybean cultivation. Seed yield can be modified by several agronomic treatments, including fertilization and inoculation. In 2016 and 2017 seed yield was significantly the highest under the effect of Inoculant 1. N fertilization at dose 60 kg ha^−1^ impacted seed yield in 2016. Also, in 2016 the interaction of tested factors was observed. The highest seed yield was obtained under application of 60 kg ha^−1^ without inoculation and Inoculant 1 with N at 30 kg ha^−1^ (Table 8).

**Table 8:** The effect of fertilization and inoculation on seeds yield (t ha^−1^).

Inoculation	N fertilization (kg ha^−1^)	2016	2017	2018	2019
Mean for factors					
Uninoculated		2.42b	2.02b	2.62a	3.58a
Inoculant 1		3.11a	2.57a	2.72a	3.70a
Inoculant 2		2.45b	2.20ab	2.70a	3.78a
	0	2.34c	2.05a	2.64a	3.61a
	30	2.67b	2.34a	2.65a	3.68a
	60	2.97a	2.40a	2.75a	3.77a
Mean for interaction					
Uninoculated	0	1.68d	1.86a	2.45a	3.53a
	30	2.32c	2.03a	2.62a	3.47a
	60	3.27a	2.16a	2.78a	3.74a
Inoculant 1	0	2.96ab	2.25a	2.75a	3.59a
	30	3.34a	2.74a	2.68a	3.78a
	60	3.02ab	2.73a	2.73a	3.73a
Inoculant 2	0	2.39c	2.05a	2.71a	3.72a
	30	2.34c	2.25a	2.65a	3.78a
	60	2.63bc	2.30a	2.74a	3.84a

Different letters indicate significant differences (Tukey’s multiple range test).

Soybean straw yield is important from an environmental and agronomic point of view. Crop residues are a source of nitrogen and when ploughed they enrich the soil with this nutrient. Below the effect of tested factors and their interaction on straw yields is shown. Please refer to the supplementary material for details (Supplemetary Table S5). The effect of inoculation was observed in 2016 and 2019. Inoculant 1 application caused higher straw yield in 2016, while in 2019 using of Inoculant 2 gave better results. N fertilization was significantly important only in 2016, when dose of 60 kg ha^−1^ was the most effective. In 2016 the interaction of Inoculant 1 and N fertilization at 30 kg ha^−1^ contributed to highest straw yield, but in 2019 the synergistic effect of Inoculant 2 and N at 60 kg ha^−1^ was the most beneficial among all tested combinations (Supplementary Table S5).

## Discussion

Many factors affect the success of soybean cultivation expecially the genetic variability, weather conditions or any agrotechnical treatments like fertilization. Soybean seed yield is dependent on climatic conditions: air temperature, total precipitation and their distribution^34^, although water deficit is the most limiting factor^7,29,35^. In most regions of Europe, soybean requires about 500 mm of precipitation during the growing season, including at least 300 mm at the period of the flowering and pod setting stages^36^. According to Stojmenova and Alexieva^37^ soybean seed yield depends mainly on rainfall totals in May, July and August, as soybean assimilates about 20% of N from the beginning of flowering, and 80% during generative development. Taking into account conditions of favorable precipitation from June to August in the third and fourth year of the study (2018, 2019), plant response to inoculation and mineral N fertilization was significantly different than in the other years (2016, 2017). According to Korsak-Adamowicz et al.^38^ neither drought nor high air temperature favor the symbiosis of soybean and *B. japonicum*, however inoculation undoubtedly increases soybean yields. Rainfall in April has a positive effect on soybean as it needs a certain amount of water to germinate when it is first planted. Studies have shown that flooding leads to a significant decrease in soybean yields, and the longer the duration of flooding, the greater the decrease in soybean yields^39,40^. Excessive rainfall in May may reduce the survival rate of soybean seedlings. In our study the highest value of seed yield was obtained in 2019 where the long period of water defict occurred during most of the year (Fig. 1). When soybean is exposed to high temperatures at the seed-filling phase, its yield is reduced and their seed composition is altered^41^. It is proved that for every 1 °C increase, yield of soybean decreases by an average of 17%^42^. Some studies have shown a decrease in yield under high temperatures at the seed-filling stage of soybean^43,44^. It has been observed that high temperature stress experienced during the mid reproductive growth is more detrimental to yield and seed size than occurring early or late in reproductive development^45^.

In our study, the highest seed yield was obtained in 2019 when favourable moisture conditions were during sowing, germination and in early vegetative development stages (April-June), while water deficit occurred during later stages. This shows that soybean is drought tolerant and needs optimal moisture conditions during the earliest growth stages for proper development and yield, when grown in countries at higher latitudes, such as Poland.

Seed inoculation is the most beneficial practice to increase soybean seed yield, especially in regions where this plant is not widely grown and soils are poor in symbiothic bacteria strains^46^as it is in the case in most European countries, including Poland. *Rhizobium* bacteria affect positively crop growth and development, as well as macro-and microelements uptaking increasing the production of plant hormones that promote crop growth^47^. *Rhizobium* is promising to be a friendly substitute for nitrogen ferttilizers^48^. *Rhizobium* form symbiotic relationships with legumes delivering in this way a significant amount of nitrogen to plants, therefore continuous inoculation ensures long-term soil nitrogen availability^49^.

Many studies^5,50,51^ have shown that the application of seed inoculation significantly increased soybean yield, compared to the control. Capatana et al.^52^ obtained an increase in soybean seed yield after inoculation, but only by 3.76% compared to the control. Mpepereki et al.^53^ showed that a properly selected variety and inoculant caused a significant increase in the soybean seed yields. Based on a study by Ulzen et al.^54^ many commercial inoculants (Biofix, Legumefix and BR 3267) can be used to increase seed yields of soybean and cowpea. In contrast, Abou-Shanab et al.^55^ presented the data that inoculation did not increase the soybean seed yield. Zainal et al.^56^ added that inoculation with nitrogen-fixing bacteria was not always successful in increasing high production. Mayani and Hapsoh^57^ also explained that the application of *Rhizobium* had no significant effect on the number of pods per plant. Our study also showed, that inoculation of soybean seeds with commercial products increased the following yield components: the number of pods per plant (2016, 2017), number of seeds per plant (2016, 2017), as well as seeds yield (2016, 2017) and straw yield (2016, 2019), compared to control. However the effect of inoculation on 1000 seed weight was not found except in 2017, which was also not observed by Szpunar-Krok et al.^26^. In contrast, the study by Panasiewicz et al.^25^ showed, that 1000 seed weight was the most affected by inoculation compared to control, among all tested yield components. In a study by Herliana et al.^58^, the application of a *Rhizobium* isolate influenced the variables pod number. In study of Yousaf et al.^59^ seed inoculation with *R. japonicum* or *P. fluorescens* caused the increase in the yield and quality of different genotypes of soybean compared to uninoculated seeds.

N fertilization is not a common practice in soybean cultivation in the world, due to biological nitrogen fixation by rhizobacteria. However soybean response to mineral nitrogen application have been investigated^60,61^. Most scientific studies indicate that nitrogen fertilization has no effect on soybean seed yield. This is because of the loss of root nodulation efficiency, which makes plants dependent on mineral fertilizers, which increases production costs^62^. According to their results, N application to soybean plants with high seed yield is positive. It turned out that regional conditions: climate, soil and weather as well as production system affect the response of soybean to N fertilization. Soybean can respond better to N application in agricultural systems where yields may be limited due to low soil fertility, water shortages and high temperatures^61^. Some studies have shown that N increases soybean seed yields, but without economic benefits^63^.

Nitrogen fertilization can contribute to increase in seed yields by allowing soybean to allocate photosynthesis products to seeds instead of biological nitrogen fixation (BNF)^64^. The response of soybean to N fertilization can vary in relation to the type and dose of fertilizer, the phase of development during application and even the method of application^65^ combining with other environmental factors^66^.

Studies conducted in Poland showed that N fertilization at dose 60 kg ha^−1^ led to significant increase in yield components values and in general to higher seed yield, compared to control^25,26,67^. However, Szpunar-Krok et al.^26^ concluded, that due to small differences in obtained yield, when comparing 60 kg N ha^−1^, 30 kg N ha^−1^ and control, the use of high doses of N is not necessary, but seed inoculation with symbiotic bacteria should be considered as a more yield-forming treatment. This is important from an environmental and economic point of view. In the experiment by Ulze et al.^54^ nitrogen supplied in the form of urea at a rate of 100 kg N ha^−1^ significantly increased seed yield. Yield increases may not be observed in soils that receive a high-quality inoculant if nitrogen is not the limiting factor^68^.

According to Simonis and Setatou^69^ research, N fertilization is not necessary because soils in Greece have a sufficient number of rhizobia coming from previous crops or seed inoculation. They reported that N fertilization at the beginning of the pod-filling stage increased yields by 3–20%. The same recommendation concerned Brazil where the use of inoculant containing *Bradyrhizobium* strains without the addition of N fertilizer is sufficient (exceptionally application of 20 kg ha^−1^ of N at sowing when the soil is poor in organic matter)^70^. In contrast, in the northern Great Plains (central United States of America and western Canada), the use of N as a starter has the potential to increase soybean yield and early plant growth^71^.

However, BNF process as well as N fertilization is seldom disrupted by abiotic stress (caused by unfavorable weather and soil conditions). Therefore, N fertilization is necessary. Scharf and Wiebold^72^ presented a response of soybean plants to N application in amount of 33 kg ha^−1^ in soils with a pH lower than 6. The same results were proved by Caliskan et al.^73^. Where an increase in soybean seed yield with N application up to 80 kg ha^−1^ in soil with a pH above 7 was observed.

Sogut et al.^74^found in the research that seed inoculation with a low nitrogen dose had a positive effect on soybean yield. For maximum yield, 30 kg of N ha^−1^ with inoculation, and 60 kg N ha^−1^ without inoculation were needed. El-Shaarawi et al.^75^ claimed the integrated application of *B. japonicum* and nitrogen improved the growth and yield of soybean, what is consistent with our results.

In our experiment, the synergistic effect of nitrogen fertilization at 30 kg ha^−1^ with Inoculant 1 in 2016 gave the highest seed and straw yield. This combination had the greatest impact on number of pods per plant (2016, 2017 jointly with the dose of 60 kg ha^−1^) and number of seeds per plant (2016, 2017) straw yield (2016), what directly led to the higest seed yield obtained. Similar conclusions were drawn by Panasiewicz et al.^25^, who found that a significant increase in seed yield was observed after nitrogen fertilization at rates of 30 kg N ha^−1^ or 60 kg N ha^−1^, combined with the application of HiStick® Soy, compared to the control. These authors concluded that due to the lack of a significant difference in yield and higher fertilizer costs, an application of 30 kg N per ha in combination with HiStick® Soy is optimal for soybean cultivation under Polish conditions. Synergistic beneficial effect of N mineral fertilization with seeds inoculation was also reported by Capatana et al.^52^, who found that soybean seed yield increased by 3.7% after inoculation alone, while mineral fertilization with inoculation increased yield by about 30%. Study by Szpunar-Krok^26^ showed that under Polish agroclimatic conditions also the mutual effect of mineral fertilization at a dose of 30 kg N per ha and inoculation, but irrespective of the inoculant used, had the most favorable effect on soybean yield and yield components.

The research showed that soybean cultivation is a broad issue and must take into account the potential for biological nitrogen fixation, supported by inoculation. It is important to realise that BNF may not be sufficient to meet plant requirements, and N fertilization may be necessary to achieve satisfactory seed yields, especially in countries where soybean cultivation is still a challenge.

Results of this study are partly consistent with work hypothesis, as the combined effect of mineral fertilization at dose 30 kg ha^−1^ and inoculation on soybean productivity was most beneficial. However, similar effects were observed when 60 kg N ha^−1^ was applied both alone and with inoculation. But, due to environmental impact and in order to fully exploit the plants' capacity for BNF, we recommend using lower doses of N fertilizer and inoculating seeds with *B. japonicum*.

## Conclusions

The results indicate that synergistic effect of nitrogen fertilization at dose 30 kg ha^−1^ with seeds inoculation with an Inoculant 1 containing *B. japonicum* had the strongest impact on seed yield and its components, among fertilization and inoculation combinations tested. The use of higher dose of mineral fertilizer is not economically justified. Recommendations for the application of inculation should be linked to regional soil conditions. The results of our research showed that it is necessary to apply a certain amount of starter nitrogen fertilizer in combination with seed inoculation during soybean cultivation in Poland in order to meet the plant requirements for proper development and yield. When applying nitrogen fertilizers, doses should be chosen to be environmentally and economically beneficial.

### Supplementary Information

Supplementary Tables.

## Data Availability

All data generated or analysed during this study are included in this published article.
