# Supplementary material for: Influence of nitrogen fertilization, seed inoculation and the synergistic effect of these treatments on soybean yields under conditions in south-western Poland
Source: Sci Rep. 2024 Mar 20;14:6672. doi: 10.1038/s41598-024-57008-y (PMC10954626; doi:10.1038/s41598-024-57008-y)
Supplement: Supplementary file 1 — Supplementary Tables. [file 41598_2024_57008_MOESM1_ESM.docx]

**Influence of nitrogen fertilization, seed inoculation and the synergistic effect of these treatments on soybean yields under conditions in south-western Poland**

**Magdalena Serafin-Andrzejewska, Anna Jama-Rodzeńska, Waldemar Helios, Marcin Kozak, Sylwia Lewandowska, Dariusz Zalewski, Andrzej Kotecki**

**Supplementary Table S1.** The effect of fertilization and inoculation on plant density (pcs m^-2^) for each year of experiment.

| Inoculation | N fertilization  (kg ha^-1^) | 2016 | 2017 | 2018 | 2019 |
| --- | --- | --- | --- | --- | --- |
| Mean for factors | | | | | |
| Uninoculated |  | 77.0a | 52.8b | 65.7a | 68.1a |
| Inoculant 1 |  | 73.7a | 51.7b | 67.7a | 67.9a |
| Inoculant 2 |  | 74.3a | 59.2a | 68.3a | 70.2a |
|  | 0 | 74.7a | 55.0a | 68.7a | 67.6a |
|  | 30 | 75.3a | 54.7a | 66.8a | 69.6a |
|  | 60 | 75.0a | 53.9a | 66.4a | 69.0a |
| Mean for interaction | | | | | |
| Uninoculated | 0 | 76.0a | 58.3ab | 69.0a | 68.0a |
|  | 30 | 77.0a | 48.4 ab | 65.3a | 66.0a |
|  | 60 | 78.0a | 51.7 ab | 62.9a | 70.3a |
| Inoculant 1 | 0 | 74.0a | 48.3b | 70.0a | 67.5a |
|  | 30 | 76.0a | 55.0 ab | 65.0a | 72.0a |
|  | 60 | 71.0a | 51.7 ab | 68.1a | 64.2a |
| Inoculant 2 | 0 | 74.0a | 58.4 ab | 67.0a | 67.3a |
|  | 30 | 73.0a | 60.8a | 70.0a | 70.8a |
|  | 60 | 76.0a | 58.3 ab | 68.0a | 72.6a |

**Supplementary Table S2.** The effect fertilization and inoculation on number of pods per plant (pcs) for each year of experiment.

| Inoculation | N fertilization  (kg ha^-1^) | 2016 | 2017 | 2018 | 2019 |
| --- | --- | --- | --- | --- | --- |
| Mean for factors | | | | | |
| Uninoculated |  | 18.4c | 15.8b | 21.1a | 24.6a |
| Inoculant 1 |  | 22.5a | 21.8a | 20.3a | 24.9a |
| Inoculant 2 |  | 19.3b | 17.6b | 20.7a | 24.9a |
|  | 0 | 17.0c | 16.1b | 20.4a | 23.5a |
|  | 30 | 20.9b | 19.3a | 20.3a | 23.9a |
|  | 60 | 22.4a | 19.7a | 21.4a | 27.0b |
| Mean for interaction | | | | | |
| Uninoculated | 0 | 11.5d | 12.9a | 20.5a | 23.9a |
|  | 30 | 19.2c | 16.4ba | 20.9a | 22.9a |
|  | 60 | 24.6a | 18.0a | 21.9a | 26.8a |
| Inoculant 1 | 0 | 20.8b | 18.3a | 20.4a | 22.9a |
|  | 30 | 25.2a | 23.5a | 20.0a | 24.4a |
|  | 60 | 21.6b | 23.5a | 20.6a | 27.2a |
| Inoculant 2 | 0 | 18.7c | 17.0a | 20.3a | 23.5a |
|  | 30 | 18.3c | 17.9a | 19.9a | 24.2a |
|  | 60 | 21.0b | 17.7a | 21.8a | 26.8a |

**Supplementary Table S3.** The effect fertilization and inoculation on number of seeds per plant for each year of experiment.

| Inoculation | N fertilization  (kg ha^-1^) | 2016 | 2017 | 2018 | 2019 |
| --- | --- | --- | --- | --- | --- |
| Mean for factors | | | | | |
| Uninoculated |  | 35.8b | 29.0b | 38.3a | 44.5a |
| Inoculant 1 |  | 45.0a | 38.0a | 37.5a | 47.2a |
| Inoculant 2 |  | 36.7b | 29.9b | 38.0a | 45.8a |
|  | 0 | 33.8c | 29.2b | 37.5a | 43.4b |
|  | 30 | 41.2b | 33.1a | 37.7a | 44.4b |
|  | 60 | 42.5a | 34.6a | 38.7a | 49.8a |
| Mean for interaction | | | | | |
| Uninoculated | 0 | 24.1g | 23.5c | 23.5a | 45.2a |
|  | 30 | 36.5ef | 30.5b | 30.5a | 40.2a |
|  | 60 | 46.8b | 32.9b | 32.9a | 48.2 a |
| Inoculant 1 | 0 | 43.6c | 34.4ab | 34.4a | 43.1 a |
|  | 30 | 50.4a | 39.5a | 39.5a | 45.8a |
|  | 60 | 41.0cd | 40.2a | 40.2a | 52.7 a |
| Inoculant 2 | 0 | 33.8f | 29.7bc | 29.7a | 41.9 a |
|  | 30 | 36.6e | 29.4bc | 29.4a | 47.1 a |
|  | 60 | 39.9d | 30.7b | 30.7a | 48.4 a |

**Supplementary Table S4.** The effect fertilization and inoculation on weight of 1000 seeds (g) for each year of experiment.

| Inoculation | N fertilization  (kg ha^-1^) | 2016 | 2017 | 2018 | 2019 |
| --- | --- | --- | --- | --- | --- |
| Mean for factors | | | | | |
| Uninoculated |  | 137a | 152b | 149a | 144a |
| Inoculant 1 |  | 145a | 168a | 156a | 147a |
| Inoculant 2 |  | 137a | 158ab | 153a | 146a |
|  | 0 | 146a | 158a | 154a | 143a |
|  | 30 | 138a | 159a | 152a | 147a |
|  | 60 | 136a | 160a | 152a | 146a |
| Mean for interaction | | | | | |
| Uninoculated | 0 | 146a | 152a | 151a | 139a |
|  | 30 | 132a | 150a | 147a | 152a |
|  | 60 | 133a | 152a | 149a | 141a |
| Inoculant 1 | 0 | 146a | 168a | 159a | 145a |
|  | 30 | 147a | 169a | 155a | 148a |
|  | 60 | 143a | 167a | 155a | 148a |
| Inoculant 2 | 0 | 145a | 155a | 153a | 145a |
|  | 30 | 134a | 157a | 154a | 142a |
|  | 60 | 133a | 162a | 152a | 151a |

**Supplementary Table S5.** The effect of fertilization and inoculation on straw yield (t ha^-1^).

| Inoculation | N fertilization  (kg ha^-1^) | 2016 | 2017 | 2018 | 2019 |
| --- | --- | --- | --- | --- | --- |
| Mean for factors | | | | | |
| Uninoculated |  | 4.43b | 2.89a | 2.97a | 2.29b |
| Inoculant 1 |  | 5.32a | 2.99a | 2.93a | 2.24b |
| Inoculant 2 |  | 4.68b | 3.13a | 2.92a | 2.66a |
|  | 0 | 4.37b | 2.91a | 2.97a | 2.30a |
|  | 30 | 4.79b | 2.90a | 2.94a | 2.35a |
|  | 60 | 5.26a | 3.19a | 2.90a | 2.55a |
| Mean for interaction | | | | | |
| Uninoculated | 0 | 3.64d | 2.97a | 2.81a | 2.45b |
|  | 30 | 4.23cd | 2.69a | 3.08a | 2.24b |
|  | 60 | 5.41ab | 3.02a | 3.02a | 2.18b |
| Inoculant 1 | 0 | 5.05abc | 2.77a | 3.02a | 2.11b |
|  | 30 | 5.65a | 3.06a | 2.86a | 2.39b |
|  | 60 | 5.25ab | 3.14a | 2.90a | 2.23b |
| Inoculant 2 | 0 | 4.42bcd | 3.00a | 3.09a | 2.33b |
|  | 30 | 4.50bcd | 2.97a | 2.87a | 2.41b |
|  | 60 | 5.11abc | 3.42a | 2.80a | 3.23a |
